# Decreased lateral posterior tibial slope and medial tibial depth are underlying anatomic risk factors for posterior cruciate ligament injury: a case–control study

**DOI:** 10.1186/s12891-022-05653-7

**Published:** 2022-07-20

**Authors:** Baoshan Yin, Pei Zhao, Jiaxing Chen, Wenlong Yan, Hua Zhang, Jian Zhang, Aiguo Zhou

**Affiliations:** 1 Department of Orthopaedics, the First Affiliated Hospital of Chongqing Medical University, Chongqing, 400016 China; 2 Chongqing Medical University, Chongqing, 400016 China

**Keywords:** Magnetic resonance imaging, Posterior cruciate ligament, Posterior tibial slope, Medial tibial depth

## Abstract

**Objectives:**

To research whether medial PTS, lateral PTS and MTD were different between the PCL injury group and the PCL intact group.

**Design:**

Retrospective case–control study, level of evidence III.

**Methods:**

Fifty patients with PCL rupture from 2015 to 2020 in our hospital, and 50 patients matched by age and sex with intact PCL were enrolled in our study. The intraclass correlation coefficient (ICC) was used to assess the reliability of each parameter. The independent t-test was conducted to identify the differences in tibial morphometric characteristics between the PCL-injured and PCL-intact individuals, including the posterior tibial slope (PTS), meniscal slope (MS), medial tibial depth (MTD). A binary logistic regression model was established to evaluate the roles of those anatomic parameters of interest play in PCL injuries.

**Results:**

The interobserver reliability of each parameter showed excellent agreement. Significant differences in the medial (*P* = .023) and lateral (*P* = .009) PTS were found between the PCL-injured group (3.68 ± 2.70 and 4.55 ± 3.19, respectively) and the controls (5.00 ± 2.73 and 6.39 ± 3.29, respectively). And the MTD was 1.98 ± 0.64 mm in the PCL-injured group and 2.37 ± 0.55 mm in the control group (*P* = 0.007). Binary logistic regression analysis showed that smaller lateral PTS and MTD were directly associated with PCL injury, with an OR of 1.17 and OR of 3.14, respectively. The medial PTS was independent to PCL injures.

**Conclusion:**

Decreased lateral PTS and MTD were underlying anatomic risk factors for PCL injury.

## Background

The main function of the posterior cruciate ligament (PCL) is to restrict the excessive posterior movement of the tibia relative to the femur, maintaining the posterior stability of knee joint [1, 2]. It was accepted that femorotibial biomechanics were influenced by the morphology of the tibial plateau and femoral condyles [3]. Biomechanically, excessive posterior tibial slope (PTS) was considered as a predisposing risk factor for ACL noncontact injury, because of its contribution to the increase of tensity for native ACL, particularly in axial compressive forces condition. In contrast, the stress on PCL would increase with smaller PTS.

As an adjunctive examination that can effectively detect cruciate ligament injury, measurements on MRI were valued by clinicians and researchers. For the risk factors of PCL injury, A smaller and more sharply angled intercondylar notch and a more flattened tibial eminence are risk factors of PCL injury [4]. Furthermore, a smaller notch width index (coronal) in women was found to be a risk factor in PCL avulsion fracture [5]. Moreover, shallow medial tibial plateau depression (MTD) reduced the limitation of relative motion of the femur over the tibia during closed chain movements [6, 7]. Similarly, anatomic abnormalities at the proximal tibia also have an impact on the PCL, the lesser PTS, the more tension the PCL suffered [8].

Fully understanding the impact of the proximal tibial morphology on PCL is important to guide the treatment and postoperative rehabilitation exercise of patients. However, such a study is lacking in clinical practice. Bony PTS in PCL injured knees measured on lateral radiographs was decreased compared with controls, and Giffin et al. [9] determined that increased PTS in PCL ruptured knees shifted the resting position of the tibia relative to the femur anteriorly. On the other hand, little information is available regarding tibial plateau anatomy measuring on magnetic resonance imaging (MRI) in patients with PCL rupture [10, 11].

Due to the insufficient knowledge in the literature, the principal aim of this study was to research whether medial PTS, lateral PTS and MTD were different between the PCL injury group and the PCL intact group. Our study not only filled the knowledge gap in the literature but also found that decreased lateral PTS and MTD were underlying anatomic risk factors for PCL injury. It may to some extend be helpful in rehabilitation after PCL reconstruction: it should be more conservative in terms of weight-bearing after PCLR in patients with shallow MTD and/or small lateral PTS angle, due to the tibia is more prone to backward movement relative to the femur. If the radical functional rehabilitation is carried out, the risk of the grafts loosening and rupture may be increased. In contrast, whether radical functional rehabilitation is appropriate in patients with larger MTD and/or shaft lateral PTS.

## Materials and methods

### Study population

Seventy six patients with PCL injury, identified in the Medical Record Registration System of our hospital from January 2015 to December 2020, were eligible for inclusion of this study. The inclusion criteria were subjects with an MRI scan in the picture archiving and communication system (PACS); the PCL rupture was identified by arthroscopy. Subjects with previous osteotomy or fracture, meniscal repair, or partial meniscectomy, which would influence the measurement of PTS and/or meniscal slope (MS); subjects with PCL avulsion fractures or ACL injuries; subjects with discoid meniscus were excluded from this study. Among the 76 patients, 16 patients with ACL injury, five patients with PCL avulsion fractures, three patients with severe menisci injury (Bucket Handle Tear) that can affect the accuracy of the MS, one patient with previous ACL reconstruction, one patient with lateral discoid meniscus were excluded from this study.

As a result, 50 patients with PCL rupture were included in the study. Among them, some concomitant injuries were presented and included partial meniscus rupture, cartilage injury, bone marrow edema, but those could not influence the accuracy of the measurements. The PCL-intact control group, matched by age and sex was built to include 50 individuals who came to the radiology department of our hospital for knee-MRI-scanning only with anterior knee pain and without a history of knee injury. And the MRI of each individual was retrospectively reviewed by two experienced orthopedists to ensure the participants’ eligibility for the study. The informed consent requirement was waived by the Institutional Review Board of the First Affiliated Hospital of Chongqing Medical University.

### MRI technique

The MRI examinations were performed within one week prior to surgical planning in patients with PCL injuries. All examinations were performed with the same 1.5 T MRI scanner (Siemens Magnetom Essenza, Germany). The coronal and sagittal planes were scanned with the T1-weighted turbo spin-echo (TSE) sequence and proton density (PD) TSE with the fat-suppressed (FS) sequence, and the axial plane was scanned with the PD-TSE-FS. The layer thickness was set to 3 mm, the slice gap was 0.5 mm, the field of view (FOV) was 160 mm, and the matrix size was 512 × 512.

### Measurements

Anatomic parameters were obtained on MRI by two observers in a blinded and randomized fashion through PACS workstation to determine inter-observer reliability. Both observers were trained in consensus in the measuring methods. The values measured by the 2 observers were averaged for the statistical analysis.

**Fig. 1:**
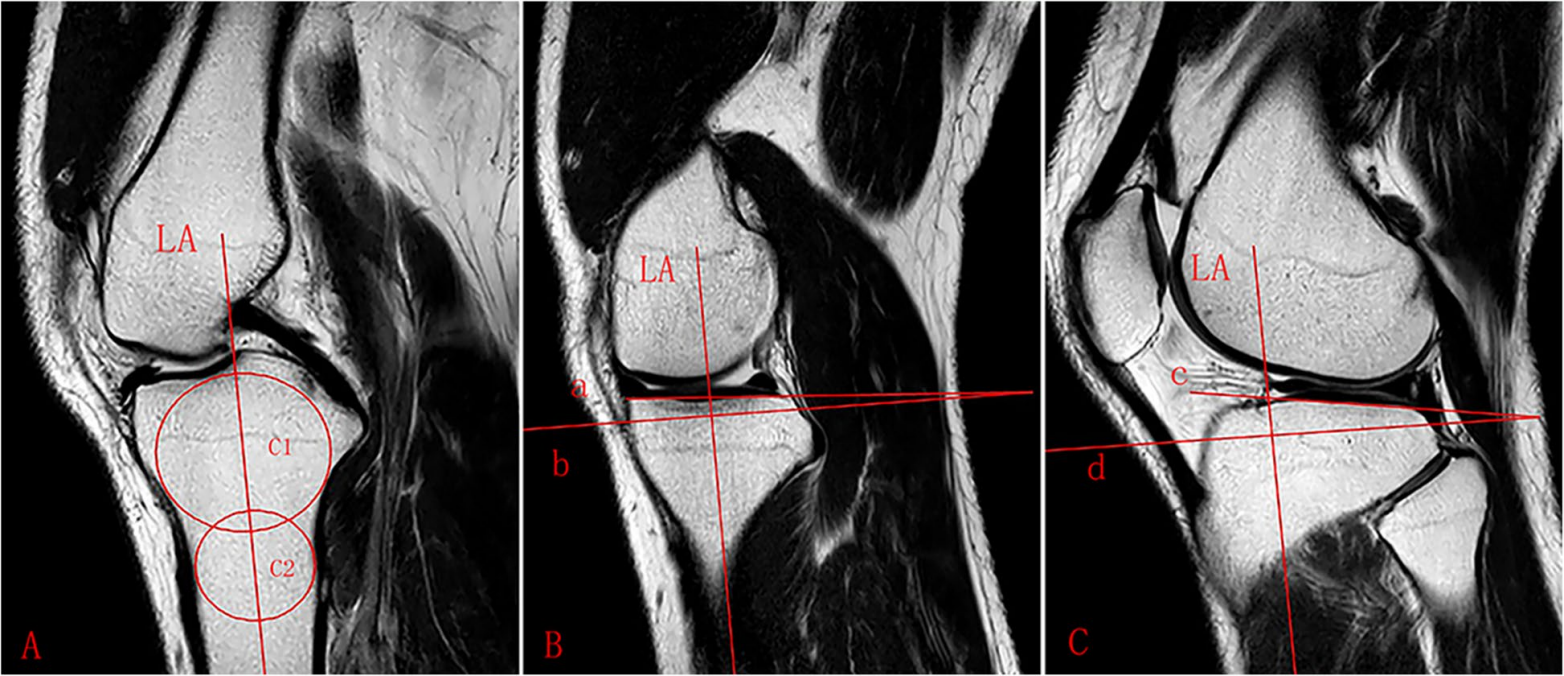
Lateral and medial PTS. **A** sagittal MRI image shows the tibial attachment of the PCL and the intercondylar eminence. C1: circle 1 has to touch the anterior, posterior, and cranial tibial cortex bone C2: circle 2 has to touch the anterior and posterior cortex border. The longitudinal axis (LA) of the proximal tibia was defined by a line that connected the centers of these two circles. **B** sagittal MRI image shows the center of the medial tibial plateau, the LA is superimposed on the selected image. Line a: the line tangent to the medial plateau. Line b: the line perpendicular to LA. The angle between line a and line b is defined as medial PTS. **C** sagittal MRI image shows the center of the lateral tibial plateau. Line c: the line tangent to the lateral plateau. Line d: the line perpendicular to LA. The angle between line c and line d is defined as lateral PTS

**Fig. 2:**
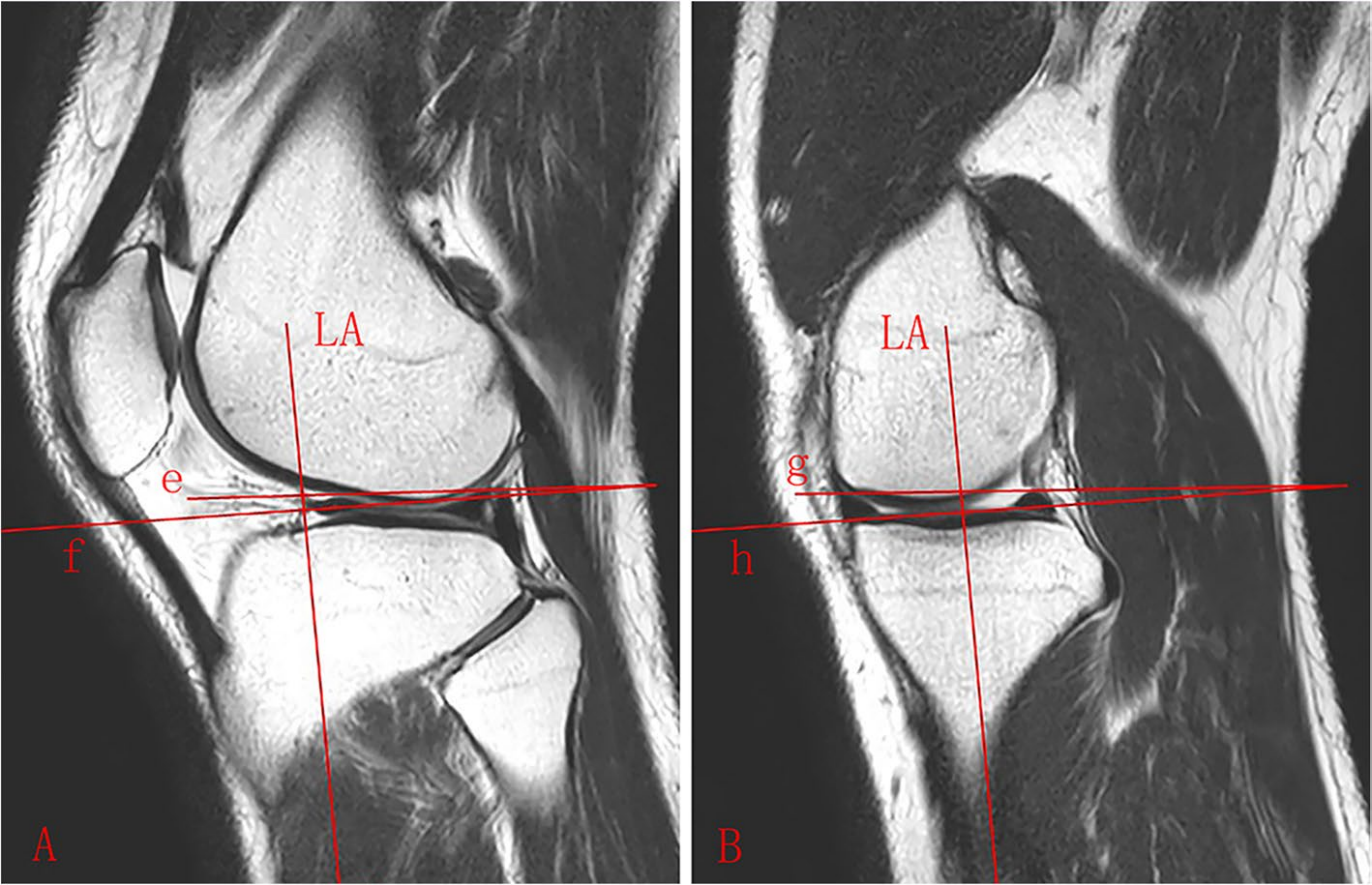
Lateral and medial MS. **A** sagittal MRI image shows the anterior and posterior horns of lateral meniscus clearly. The LA is the longitudinal axis of the proximal tibia, line f is perpendicular to LA, and line e joints the highest points of the anterior and posterior horns of lateral meniscus. The lateral MS is calculated as the angle between line e and line f. **B** sagittal MRI image shows the anterior and posterior horns of medial meniscus. Line h is perpendicular to LA, and line g joints the highest points of the anterior and posterior horns of medial meniscus. The medial MS is calculated as the angle between line g and line h

The literature suggested that separate assessment of the PTS was not reliably possible on lateral radiographs, the reliable and reproducible methods conducted on MRI or computed tomography (CT) images were recommended to measure the PTS [12]. It varies in the literature regarding the definition of the lateral tibial axis on MRI [13, 14], we conducted the method described by Hudek et al. [15], which was reported to be the most repeatable and reliable method to measure sagittal tibial slopes on MRI [13]: a T1-weighted mid-sagittal cut was selected with the appearance of PCL-tibial insertion point, the tibial axis was defined as the line through both center of circles drawing on the sagittal image, and tibial axis was used to measure the medial and lateral PTS (Fig. 1) and MS (Fig. 2) on the other two sagittal images (the mid-medial tibial plateau cut and the mid-lateral tibial plateau cut), respectively.

**Fig. 3:**
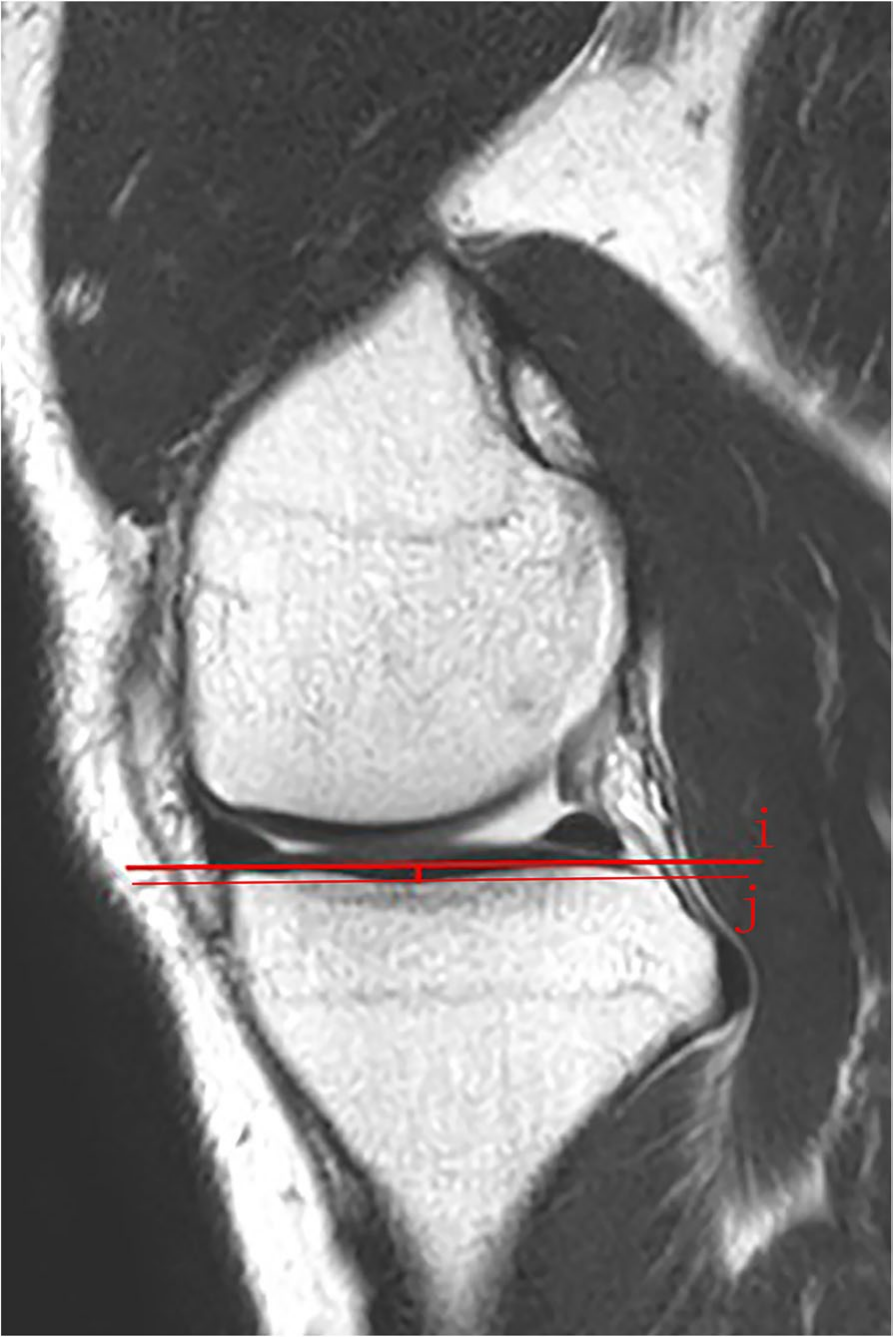
Medial tibial depth. Line i: the line tangent to the medial plateau, which is drawn to the proximal cortex border of the tibial plateau. Line j gets through the lowest point of the medial concavity and parallel to line i. The distance between the two lines is defined as medial tibial depth (MTD)

MTD is the depth of concavity of the medial plateau in the middle of the articular region. The perpendicular distance between the line connecting the uppermost superior-anterior and posterior cortex edges of the medial tibial plateau, and the lowest point of the medial plateau concavity [16] (Fig. 3).

Approval from the hospital ethics committee was obtained before this study started (IRB Number: 2021–038).

### Statistical analysis

The average values of the variables measured by both observers were used for all analyses. The Independent t-test was conducted to compare the parameters between the two subgroups, and binary logistic regression analysis was used to determine the probability of PCL injury in an individual based on the measured covariates. All analysis was conducted by a coauthor independently via the SPSS software (Version 22.0; IBM Corp), and the *P* value of < 0.05 was considered statistic significant. The interclass correlation coefficient (ICC) was calculated to assess the reliability of each parameter, with a value of more than 0.75 indicating excellent agreement.

G*Power 3.1 (Heinrich-Heine-Universitat Dusseldorf, Dusseldorf, Germany) was used to perform power analysis. Regarding the difference in the lateral PTS between the two groups, for a large effect size (0.56), the results indicated that at least 40 patients were needed in this study (alpha, 0.05; power, 0.8).

## Results

The study, as a result, included 50 patients with PCL injuries, with a mean ± standard deviation (SD) age of 43.4 ± 11.8 years, and 50 age and sex matched individuals (age of 42.7 ± 10.2) with intact PCL. The interval time between injury and MRI examination in PCL injured group was 4.2 ± 2.5 days. The descriptive data of both groups is presented in Table 1.

**Table 1:** Demographic data of the two groups

	PCL injured (*n* = 50)	Controls (*n* = 50)	*P* value
Age, yr	43.4 ± 11.8	40.7 ± 7.2	n.s
Sex, (female/male)	17/33	20/30	n.s
Side, (left/right)	20/30	23/27	n.s
BMI, kg/m^2^	25.8 ± 2.1	24.9 ± 2.8	n.s
Interval time, day	4.2 ± 2.5		

*PCL* Posterior cruciate ligament, *BMI* Body mass index, Interval time, the mean time between injury and MRI examination

The interobserver reliability of each parameter both in the PCL injured group and control group showed excellent agreement, the ICC of each measurement was more than 0.75 (Table 2). The results of the independent t-test for comparison of the variables between the two groups were presented in Table 3. Significant differences in the medial (*P* = 0.023) and lateral (*P* = 0.009) PTS were found between the study group (3.68 ± 2.70 and 4.55 ± 3.19, respectively) and the control group (5.00 ± 2.73 and 6.39 ± 3.29, respectively). The MTD was 1.98 ± 0.64 mm in the PCL-injured group and 2.37 ± 0.55 mm in the control group (*P* = 0.007). Both medial MS and lateral MS were smaller in the PCL-injured group than those in the control group, but the differences did not show statistical significance (*p* = n.s.).

**Table 2:** Inter-observer reliability analysis and mean ± SD of PTS, MS, MTD in two subgroups

Variables	PCL ruptures group				Control group			
	Observer1	Observer2	Average	ICC (95% CI)	Observer1	Observer2	Average	ICC (95% CI)
PTS								
Lateral PTS	4.53 ± 3.36	4.58 ± 3.06	4.55 ± 3.19	0.985 (0.973, 0.992)	6.52 ± 3.44	6.26 ± 3.24	6.39 ± 3.29	0.968 (0.942, 0.982)
Medial PTS	3.56 ± 2.85	3.63 ± 2.65	3.68 ± 2.70	0.985 (0.972, 0.992)	4.63 ± 2.83	5.37 ± 2.87	5.00 ± 2.73	0.908 (0.832, 0.949)
MS								
Lateral MS	2.60 ± 3.23	2.87 ± 3.42	2.74 ± 3.29	0.977 (0.957, 0.987)	2.90 ± 2.98	3.24 ± 3.41	3.07 ± 3.08	0.919 (0.852, 0.955)
Medial MS	3.28 ± 3.83	3.41 ± 3.33	3.34 ± 3.52	0.960 (0.926, 0.978)	3.99 ± 2.90	4.26 ± 3.31	4.13 ± 3.00	0.923 (0.860, 0.958)
MTD	2.00 ± 0.62	1.96 ± 0.69	1.98 ± 0.64	0.924 (0.861, 0.959)	2.31 ± 0.52	2.44 ± 0.62	2.37 ± 0.55	0.902 (0.822, 0.946)

*PTS* Posterior tibial slope, *MS* Meniscus slope, *MTD* Medial tibial depth, *ICC* Intraclass correlation coefficient, *SD* Standard deviation, *CI* Confidence interval, ICC with a value more than 0.75 indicates excellent agreement

**Table 3:** Comparison of the variables between subgroups

Variables	PCL ruptures	Controls	*P* value
PTS			
Lateral PTS	4.55 (-0.3, 11.54)	6.39 (0.1, 13.17)	0.009*
Medial PTS	3.68 (-3.10, 9.88)	5.00 (-0.32, 12.39)	0.023*
MS			
Lateral MS	2.74 (-5.72, 11.53)	3.07 (-4.28, 9.94)	0.513
Medial MS	3.34 (-6.38, 11.28)	4.13 (-2.15, 11.10)	0.193
MTD	1.97 (1.10, 3.05)	2.37 (0.95, 3.75)	0.007*

*PTS* Posterior tibial slope, *MS* Meniscus slope, *MTD* Medial tibial depth

Showing by the results of Binary logistic regression analysis (Table 4), as eliminating other parameters from the regression model (simple analysis), lateral PTS and medial PTS revealed a significant OR of 1.19 (95% CI [1.04, 1.37]) and OR of 1.20 (95% CI [1.11, 1.42]) with regard to PCL injury, respectively; the MTD was associated with PCL injury with an OR of 3.20 (95% CI [1.42, 7.19]); Medial MS was not considered as risk factors for PCL injuries (*P* > 0.05). While the values of other parameters were considered constant (multivariable analysis), it was fond that except medial PTS, decreased lateral PTS and MTD were risk factors for PCL injury, with an OR of 1.17 (95% CI [1.01, 1.37]) and 3.14 (95% CI [1.28, 7.73]), respectively, indicating that for 1 mm decrease in MTD and 1 degree decrease in lateral PTS, the risk of PCL injury will increase 3.1 times and 1.2 times, respectively.

**Table 4:** Binary logistic regression model of parameters of interest for PCL injury

Variables	Unadjusted^a^			Adjusted^b^		
	OR	95% CI	*P* value	OR	95% CI	*P* value
Lateral PTS	1.19	1.04–1.37	0.011*	1.17	1.01–1.37	0.038*
Medial PTS	1.20	1.11–1.42	0.029*	1.20	0.95–1.52	0.131
MTD	3.20	1.42–7.19	0.005*	3.14	1.28–7.73	0.013*
Medial MS	1.09	0.95–1.25	0.193	0.98	0.80–1.20	0.838

*PTS* Posterior tibial slope, *MS* Meniscus slope, *MTD* Medial tibial depth, *CI* Confidence interval, *OR* Odds ratio; ^a^simple logistic analysis of the parameters *P* < 0.2 for t test; ^b^multivariable logistic analysis; *statistically significant *P*-value (*P* < 0.05)

## Discussion

The most inspiring findings of the present study were that both PTS and MTD were smaller in patients with PCL injuries than those in controls, but the MS between the two groups was not statistically different. And except the medial PTS, the lateral PTS and MTD were considered underlying anatomic risk factors for PCL injury.

As for anatomic parameters in patients with PCL injury, recent literature [13] proposed that PTS measured on lateral radiographs was decreased as compared with PCL-intact controls, but to the best of our knowledge, the medial and lateral PTS measured on MRI were unclear. As depicted by Bernhardson et al. [17], PCL graft forces increased as a small tibial slope presented after PCL reconstruction. In addition, Gwinner et al. [18] suggested that PTS could influence the stability of the knees, especially in patients with flattened PTS. In accordance with previous researches, the medial and lateral PTS were found smaller in patients with PCL-injures when compared to controls in this study. The difference in MS between subgroups in our study did not show statistical significance, indicating that soft tissue had some compensatory effect on bony PTS. Soft tissues (meniscus or cartilage) contributed to formulating a restriction of posterior direction motion on the knee joints. 3/76 (3.9%) patients in this study were accompanied by severe menisci injury, which was an important manifestation of the loss of soft tissue restriction after PCL injury. It was unclear whether the front of the tibial plateau was thicker or the rear was thinner, further researches should focus on this issue.

On the other hand, there was a controversy in the literature whether the MS in patients with ACL injury was higher compared with controls[19–21]. The concavity of medial tibial plateau was considered as a kind of stabilization of knee joints, and it was found decreased in patients with ACL injury [22]. In this study, the difference in MS between subgroups did not show statistical significance, and MTD was significantly decreased in patients with PCL injuries compared with controls, which was in accordance with previous studies related to the ACL injury [14, 19, 20]. Bernhardson et al. [17] found that in patients with posterior cruciate ligament reconstruction, the forces of PCL grafts increased with the decrease of PTS, and the increased PTS was the protection of PCL reconstruction grafts.

We also identified that decreased lateral PTS and MTD were risk factors for PCL injury. It suggested that in patients with PCL injury, bony structures (PTS and MTD) played an important role in limiting the forward and backward movement of the tibia related to the femur in patients with PCL injuries. Shelburne et al. [7] modeled cruciate force and found that increase in PTS decreased the native PCL force, noting an increase in PCL force when the slope was decreased. Patients with smaller PTS could suffer higher stress on their PCL, making it more susceptible to injury with lesser violence.

Medial PTS was significantly decreased in patients with PCL injury compared with PCL intact controls, but it was found not a risk factor for PCL injury. The medial tibial plateau mainly affects the stability of the knee joint in the anteroposterior direction, while the lateral tibial plateau is mainly related to the stability of the knee joint rotation. This may indicate that PCL injury is closely related to the knee joint rotation instability. Amis et al. [23] found that in patients with posterior cruciate ligament injury, tibial posterior draw was minimal when the knee was extended, and increased with knee flexed. When the knee was intact, a posterior draw force caused tibial external rotation, which can be reduced or eliminated by cutting the PCL, this suggested that PCL to some extend plays an important role in external rotation of the knee joint. Our works emphatically from the imaging aspects, studied the medial PTS and lateral PTS in patients with PCL injury, found that the lateral PTS obviously smaller compared to PCL intact controls. A number of studies have shown that the smaller the PTS, the greater the force on PCL, it means PCL in its anterior and posterior stabilities was influenced by PTS.

The result of this study may to some extend be helpful in rehabilitation after PCL reconstruction: it should be more conservative in terms of weight-bearing after PCLR in patients with shallow MTD and/or small lateral PTS angle, due to the tibia is more prone to backward movement relative to the femur. If the radical functional rehabilitation is carried out, the risk of the grafts loosening and rupture may be increased. In contrast, whether radical functional rehabilitation is appropriate in patients with larger MTD and/or shaft lateral PTS. The cutoff values of lateral and medial PTS and MTD were unclear.

We acknowledged some limitations to this study. Injury mechanisms (contact and non-contact) and concomitant injuries (medial or fibular collateral ligaments) are not included in the analysis, which may influence the importance of the tibial slope and depth. Some femoral or tibial anatomic parameters are not included in this study, such as lateral femoral condyle ratio [24], the shape of femoral notch [25], lateral tibial plateau height. Further research, including femoral morphology is warranted. Notwithstanding these deficiencies, the study has addressed the gap in understanding tibial morphology in patients with PCL injury, and identifies the underlying risk factors for PCL injury. Future researches on anatomic parameters of femoral condyle are required in patients with PCL ruptures.

## Conclusion

Decreased lateral PTS and MTD were underlying anatomic risk factors for PCL injury.

## Data Availability

The datasets analysed during the current study are not publicly available due to ethical restrictions, but are available from Aiguo Zhou on reasonable request at zhouaiguo@hospital.cqmu.edu.cn.

## Abbreviations

PCL: Posterior cruciate ligament
ICC: Intraclass correlation coefficient
PTS: Posterior tibial slope
MS: Meniscal slope
MTD: Medial tibial depth
ACL: Anterior cruciate ligament
MRI: Magnetic resonance imaging
PACS: Picture archiving and communication system
TSE: Turbo spin-echo
PD: Proton density
FS: Fat-suppressed
FOV: The field of view
CT: Computed tomography
